# Molecular Phylogeny and Biogeographic History of the Armored Neotropical Catfish Subfamilies Hypoptopomatinae, Neoplecostominae and Otothyrinae (Siluriformes: Loricariidae)

**DOI:** 10.1371/journal.pone.0105564

**Published:** 2014-08-22

**Authors:** Fábio F. Roxo, James S. Albert, Gabriel S. C. Silva, Cláudio H. Zawadzki, Fausto Foresti, Claudio Oliveira

**Affiliations:** 1 Laboratório de Biologia e Genética de Peixes, Departamento de Morfologia, Universidade Estadual Paulista, UNESP, Botucatu, SP, Brazil; 2 Department of Biology, University of Louisiana at Lafayette, Lafayette, Louisiana, United States of America; 3 Nupélia, Universidade Estadual de Maringá, UEM, Maringá, PR, Brazil; Field Museum of Natural History, United States of America

## Abstract

The main objectives of this study are estimate a species-dense, time-calibrated molecular phylogeny of Hypoptopomatinae, Neoplecostominae, and Otothyrinae, which together comprise a group of armoured catfishes that is widely distributed across South America, to place the origin of major clades in time and space, and to demonstrate the role of river capture on patterns of diversification in these taxa. We used maximum likelihood and Bayesian methods to estimate a time-calibrated phylogeny of 115 loricariid species, using three mitochondrial and one nuclear genes to generate a matrix of 4,500 base pairs, and used parametric biogeographic analyses to estimate ancestral geographic ranges and to infer the effects of river capture events on the geographic distributions of these taxa. Our analysis recovered Hypoptopomatinae, Neoplecostominae, and Otothyrinae as monophyletic with strong statistical support, and Neoplecostominae as more closely related to Otothyrinae than to Hypoptopomatinae. Our time-calibrated phylogeny and ancestral-area estimations indicate an origin of Hypoptopomatinae, Neoplecostominae, and Otothyrinae during the Lower Eocene in the Atlantic Coastal Drainages, from which it is possible to infer several dispersal events to adjacent river basins during the Neogene. In conclusion we infer a strong influence of river capture in: (1) the accumulation of modern clade species-richness values; (2) the formation of the modern basin-wide species assemblages, and (3) the presence of many low-diversity, early-branching lineages restricted to the Atlantic Coastal Drainages. We further infer the importance of headwater stream capture and marine transgressions in shaping patterns in the distributions of Hypoptopomatinae, Neoplecostominae and Otothyrinae throughout South America.

## Introduction

A central aim of research in modern historical biogeography is to understand the distributions of species and ecosystems in light of historical processes that shape landscape evolution [1], [2]. This effort has made rapid progress over the past decade in the study of Neotropical freshwater fishes. The continental fishes of tropical South America represent about one fifth of the world's fish species, or 10% of all vertebrate species [3], [4]. The evolutionary and ecological reasons for the origins and maintenance of this high diversity remain poorly understood. However, for obligate freshwater organisms, river capture is an important landscape-level process that can isolate lineages and promote diversification [5]–[7] by changing patterns in the connectivity of adjacent river basins [8]–[11].

River capture (also called stream capture or stream piracy) is a geomorphological process by which the flow of part of a stream or river drainage basin is diverted into that of a neighbouring basin. River capture may arise from the influence of several geomorphological processes, including tectonic uplift or tilting, damming by the actions of glaciers or landslides, denudation of watershed margins by erosion, or avulsion of watershed margins by sediment accumulation in fans and estuaries [12]–[16]. The process of headwater or lateral watershed erosion has the remarkable effect of simultaneously separating portions of river basins that were formerly connected (i.e., vicariance), and of connecting portions of river basins that were formerly isolated (i.e., geodispersal). In other words, river capture moves the physical location of watershed barriers [7], [17]. For obligate aquatic taxa, such as freshwater fishes, amphibians, and other animal and plant groups that inhabit riparian or floodplain habitats, river capture facilitates the dispersal of species between adjacent drainage basins.

The biogeographic consequences of river capture can be profound for species that are restricted to river basins, and for which watershed boundaries strongly limit dispersal [17]–[19]. As in all vicariance events, the separation of formerly adjacent river basin segments promotes allopatric divergence, and may ultimately lead to speciation. However, in addition, and unlike many vicariance-only events, *river capture always results in both the separation and the merging of adjacent river basin segments* [4], [20]. In other words, in the special case of river capture, vicariance and geodispersal are near simultaneous and complementary biogeographic processes. Further, both vicariance and geodispersal may result in concordant biogeographic patterns among many lineages that constitute a regional biota, (see fig. 10 in Lieberman [21], [22]). Importantly, in the case of river capture, and under the widely-used convention that geographic range is defined in terms of the river basin(s) a species occupies (e.g. [23], [24]), geodispersal can result in geographic range expansion without necessarily involving biotic dispersal [25].

Among Neotropical freshwater fishes, members of the family Loricariidae, armoured catfishes, represent an excellent model to study the effects of landscape evolution on lineage diversification. Loricariids inhabit most aquatic habitats and geographic regions of tropical South and Central America. About 869 loricariid species are currently recognized as valid [26], which makes this taxon the second-most species-rich family of Neotropical freshwater fishes (after Characidae). Loricariids also exhibit a broad range of ecological tolerances and geographic distributions. Many species are extreme habitat or trophic specialists [27]–[31], and many species are highly endemic, with small geographic ranges [16], [22], [33].

Within the Loricariidae the three subfamilies, Hypoptopomatinae, Neoplecostominae and Otothyrinae, have long been recognized together as natural group using morphological and molecular data [32], [34]–[38]. Separately, these three subfamilies were hypothesized to form monophyletic groups in Chiachio et al. [36] using molecular data. No formal infrafamily name has yet been applied to this clade, which we will refer to here as the HNO-clade. Each of these three subfamily-level clades is also species-rich, and the HNO-clade as a whole has 182 nominal species currently recognized [39]. Each of these subfamilies exhibits a wide geographic distribution throughout tropical cis-Andean South America, and has a lengthy and complex taxonomic history, including studies using both morphological and molecular datasets [32], [36]–[38], [40]–[49].

Here, we present a time-calibrated phylogenetic analysis of the loricariid catfish subfamilies Hypoptopomatinae, Neoplecostominae and Otothyrinae, using a combination of three mitochondrial and one nuclear gene markers, and the most species-dense taxon sampling of these groups to date. We then use parametric biogeographic methods to estimate ancestral geographic ranges, and to document several historical river-capture events in the region of Southeastern Brazil. Our results highlight the special role of river capture in the formation of the modern species richness and geographic distributions of the Hypoptopomatinae, Neoplecostominae and Otothyrinae.

## Materials and Methods

### Taxon Sampling

*Diplomystes mesembrinus* (Diplomystidae) was used as a distant outgroup to root all phylogenies. Diplomystidae has been alternatively been reported as the sister group to all other catfishes, or as the sister group to Siluroidea, a clade of catfishes that excludes Loricarioidea [50]–[56]. Additionally, samples of *Corydoras imitator*, *Corydoras oiapoquensis, Hoplosternum littorale, Callichthys callichthys* (Callichthyidae), *Astroblepus* sp. 1 and *Astroblepus* sp. 2 (Astroblepidae), *Hemipsilichthys gobio*, *H. papillatus*, *Delturus parahybae* (Loricariidae, subfamily Delturinae), *Rineloricaria lanceolata*, *Spatuloricaria* sp. 1 (Loricariidae, subfamily Loricariinae), *Hypostomus ancistroides*, *H. nigromaculatus* and *H. microstomus* (Loricariidae, subfamily Hypostominae) were included in the analysis as additional outgroups. We included in the analysis 155 specimens representing 115 loricariid species (see table S1 to all species names, localities and deposits in museums and table S2 to taxonomic summary of ingroup species).

### Ethics Statement

All fishes collected for this study were collected in accordance with Brazilian laws, under a permanent scientific collection license in the name of Dr. Claudio Oliveira (SISBIO). Additionally, our laboratory has special federal permission to keep animals and tissues from a public collection under our care. To work with the animals, we follow all the ethical prescriptions stated by our internal committee of ethic called “Comissão de Ética na Experimentação Animal” (CEEA) involving animal experiments (protocol number 388) that approved this study. After collection, animals were anesthetized with benzocaine, and a piece of muscle tissue was extracted from the right side of the body and preserved in 95% ethanol. Voucher specimens were fixed in 10% formalin for two weeks, and then transferred to 70% ethanol for permanent storage.

Vouchers of all samples were deposited in the collection of the Laboratório de Biologia e Genética de Peixes (LBP), Departamento de Morfologia, Instituto de Biociências, Universidade Estadual Paulista, Botucatu, São Paulo, Brazil, Museu de Ciências e Tecnologia, Pontifícia Universidade Católica do Rio Grande do Sul (MCP), Porto Alegre, Rio Grande do Sul, Brazil; Núcleo de Pesquisas em Limnologia, Ictiologia e Aquicultura (NUP), Universidade Estadual de Maringá, Paraná, Brazil, or the Museum of Natural History of the City of Geneva (MHNG), Geneva, Switzerland.

### DNA Extraction and Sequencing

Total DNA was extracted from ethanol preserved muscle samples with the DNeasy Tissue Kit (Qiagen), following manufacturer's instructions. Partial sequences of the genes 16S rRNA [57], cytochrome *b* (Cytb) [58], cytochrome c oxidase subunit I (COI) [59] and F-reticulon 4 [36] were amplified using polymerase chain reaction (PCR) with the primers described in Table S3. Amplifications were performed in a total volume of 12.5 µl with 1.25 µl of 10× buffer (10 mM Tris-HCl+15 mM MgCl2), 0.5 µl dNTPs (200 nM of each), 0.5 µl each 5 mM primer, 0.05 µl Platinum *Taq* Polymerase (Invitrogen), 1 µl template DNA (12 ng), and 8.7 µl ddH2O. The PCR reactions consisted of 30–40 cycles, 30 s at 95°C, 15–30 s at 48–58°C (according to primer and species), and 45–90 s at 72°C (according to gene primers). Nested-PCRs were used to amplify the nuclear marker; the first amplification was performed using the primers Freticul4-D and Freticul4-R with a total volume of 12.5 µl for 30–40 cycles (30 s at 95°C, 30 s at 48°C, and 135 s at 72°C); the second amplification was performed using the primers Freticul4 D2 and Freticul4 R2 with a total volume of 12.5 µl for 30–40 cycles (30 s at 95°C, 30 s at 53–54°C, and 135 s at 72°C). All PCR products were first visually identified on a 1% agarose gel and then purified using ExoSap-IT (USB Corporation) following instructions of the manufacturer. The purified PCR products were sequenced using the “Big DyeTM Terminator v 3.1 Cycle Sequencing Ready Reaction Kit” (Applied Biosystems), purified again by ethanol precipitation and loaded on an automatic sequencer 3130-Genetic Analyzer (Applied Biosystems) in the Instituto de Biociências, Universidade Estadual Paulista, Botucatu, São Paulo.

### Sequence and Phylogenetic Analysis

All individual sequences for each species were initially analysed using the software program BioEdit 5.0.9 [60] and consensus sequences were obtained. All sequences for each gene were independently aligned using MUSCLE [61] under default parameters and the alignments inspected by eye for any obvious misalignments. Only alignment errors were corrected, where indels of 1 bp were added to introns of the reticulon gene. A quality control step was included in our workflow to detect potential cases of sequencing errors due to contamination or paralogy. Alignments for each gene were initially analysed by maximum likelihood (ML) [62] using the web servers RAxML BlackBox [63] for a previous phylogenetic analysis, and controlling potential sequencing errors involving pseudogenes, paralogous copies or even laboratory cross-contamination or mistakes during the sequencing process. After that, sequences of all genes were concatenated to perform all phylogenetic and biogeography analysis (e.g. all four genes for all specimens).

Sequences that were found misplaced in the resulting gene tree (as, for example, species of one subfamily grouped with species of an obviously non-related subfamily) were re-sequenced. Nucleotide variation, substitution patterns, and genetic distances were examined using MEGA 5.0 [64]. To evaluate the occurrence of substitution saturation for each gene separately, we estimated the index of substitution saturation (Iss) as described by Xia et al. [65] and Xia and Lemey [66] and the rate of transitions/transversions, both evaluated in the software DAMBE 5.2.31 [67]. The Iss estimation was performed without taking into account gaps because unresolved sites reduce the ability of the method to test for phylogenetic signal. The best-fit partitioning schemes and the best nucleotide evolution model for each partition were evaluated in the software PartitionFinder [68] under the information-theoretic measure of Akaike Information Criterion (AICc).

Maximum likelihood analyses were performed using RAxML Web-Servers [62]. RAxML implements a faster algorithm of heuristic searches with bootstrap pseudoreplicates (RBS). Bootstrap (BS) resampling [69] was applied to assess support for individual nodes using 1,000 replicates. Random starting trees were used for each independent ML tree search and all other parameters were set on default values. The ML analyses were conducted under different models for each partition of the matrix as evaluated for the software PartitionFinder [68] (Table S4). Bayesian inference (BI) [70] was performed evaluating alternative tree topologies through the estimation of posterior probabilities (P) using MrBayes v.3.0 [71]. The Bayesian inference was conducted under different models for each partition of the matrix as evaluated for the software PartitionFinder [68] (Table S4). The ML tree was used as a starting tree for the Markov chain Monte Carlo searches. Eight chains were run simultaneously for 100,000,000 generations and every 1000th generation a tree was sampled. The above analysis was performed twice. The distribution of log-likelihood scores was examined to determine stationary phase for each search and to decide if extra runs were required to achieve convergence, using the program Tracer 1.5 [72]. All sampled topologies beneath the asymptote (25,000,000 generations) were discarded as part of a burn-in procedure, and the remaining trees were used to construct a 50% majority-rule consensus tree in Paup* [73].

Alternative tree topologies were evaluated in the program Treefinder [74] using the Shimodaira and Hasegawa (SH) test [75], the Approximately Unbiased (AU) test [76], and the Expected Likelihood Weights (ELW) method [77]. All tests were conducted under ML with a 14 partition scheme and with the same model implemented in RAxML analysis (Table S4).

### Time Calibration and Ancestral-area estimation

The uncorrelated relaxed molecular clock (lognormal) was estimated using BEAST v.1.7.5. All clade-age estimates are presented as the mean and 95% highest posterior density (HPD) values, which are the upper and lower bounds of the HPD interval. We included two calibration points to constrain divergence dates for the 154 clades identified in our phylogenetic tree. The first calibration point was implemented as a normally-distributed prior, with an offset of 125 million years ago (Mya), and a standard deviation of 15 million years. These date-estimate parameters were selected to match current knowledge of the timing of siluriform origins and were implemented in the root of the tree. Information from the stratigraphic record and geographic distributions of living taxa indicate an origin for Siluriformes as a whole during the Lower Cretaceous (145–100 Mya; [56], [78], [79]).

The second calibration point was implemented using a log-normal prior offset to 55 Mya with a mean and standard deviation of 1 for the origin of the genus *Corydoras* lineage (node including *Corydoras imitator*, *Corydoras oiapoquensis*, *Hoplosternum littorale* and *Callichthys callichthys*). The oldest known callichthyid fossil, *Corydoras revelatus* [80] was dated by Marshall et al. [81] as Paleocene. This prior assumed 55 Mya as a minimum age. We used a macroevolutionary Birth–Death model for the diversification likelihood values and a starting tree obtained from the RAxML analysis. The analyses were conducted under different models of molecular evolution for each partition of the data matrix as evaluated by the software PartitionFinder [68] (Table S4). The ML tree obtained was used as a starting tree for the MCMC searches. The analysis was run for 50 million generations and sampled every 1000th generation. Stationarity and sufficient mixing of parameters (ESS>200) was checked using Tracer v1.5 [72]. A consensus tree was built using TreeAnnotator v1.7.5 [82].

Data on the geographic distributions of species in each of the three subfamilies analysed here (Hypoptopomatinae, Neoplecostominae and Otothyrinae) were taken from the original species descriptions and information available at catalog of Eschmeyer [26]. We assigned taxa to geographic areas using the ecoregion classifications of Vari and Malabarba [3] and Chiachio et al. [36], within the following five biogeographic regions: A, Atlantic Coastal Drainages of Southeastern Brazil; B, Upper Paraná Basin; C, Paraguay, Lower Paraná and Uruguay basins; D, Amazon and Orinoco basins; E, São Francisco basin and Coastal Drainages of Northeastern of Brazil.

A maximum-likelihood analysis of biogeographic history was also performed in Lagrange v2.0 [(Ree et al., 2005; Ree and Smith, 2008) using a DEC model of geographic range evolution. The DEC model specifies instantaneous transition rates between discrete distribution areas along the branches of a phylogenetic tree, and uses these rates to assess the likelihoods of ancestral distributions at cladogenetic events [83], [84]. Four DEC models were tested to estimate distribution ranges inherited by the descending lineages at each node of the tree. The differences between the models are in the rate of dispersal among adjacent and no adjacent areas (see Table S5 for the likelihood values and dispersal rate among adjacent and no adjacent areas for each model). The model that obtained the highest ML values was model 3 (M3) that constrained the dispersal rates between adjacent areas at 0.5 and areas separated by one or more intercalated areas at 0.0001.

## Results

### Phylogenetic Analysis

Partial sequences of three mitochondrial genes (16S rRNA, COI, Cytb) and one nuclear gene (F-reticulon 4) were obtained from 155 specimens representing 115 loricariid species (Table S1). The combined sequence data resulted in a matrix of exactly 4,500 base pairs (bp), of which 1,482 bp (33%) were non-variable (conserved), 2,677 bp (59%) were variable and included in the analysis, and 341 bp (8%) were variable indels excluded from the analysis. This matrix was used to perform all phylogenetic and biogeographic analyses and was partitioned by gene and coding positions into 14 sections (Table S4). These data were not saturated considering that the Iss.c value is greater than the Iss, and the R^2^ value is greater than 0.70 for transitions and transversions for all the genes (Table S6).

**Figure 1:**
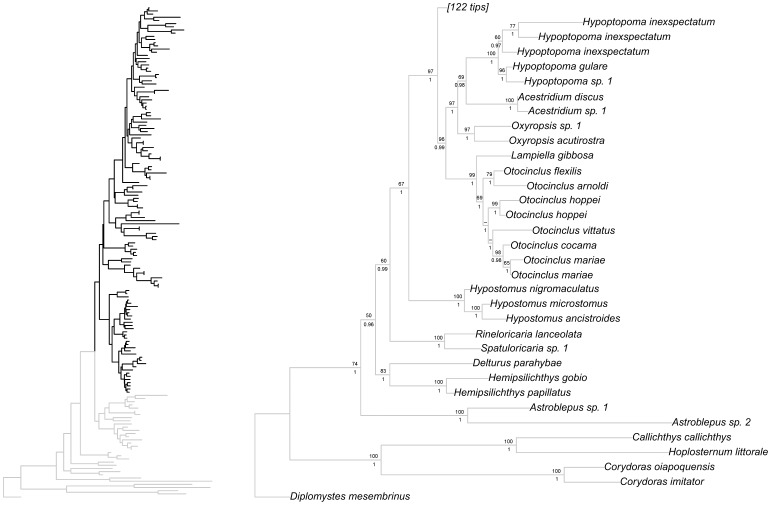
Partial ML tree showing outgroups and interrelationship among species of the subfamily Hypoptopomatinae. Numbers above branches are bootstrap values from 1000 bootstrap pseudoreplicates obtained from ML analysis. Bootstrap values below 50% (−) are not shown. Numbers below branches are posterior probabilities obtained in the BI analysis. Posterior probabilities values below 0.95 (−) or when the nodes were not obtained by B analyses are not shown.

**Figure 2:**
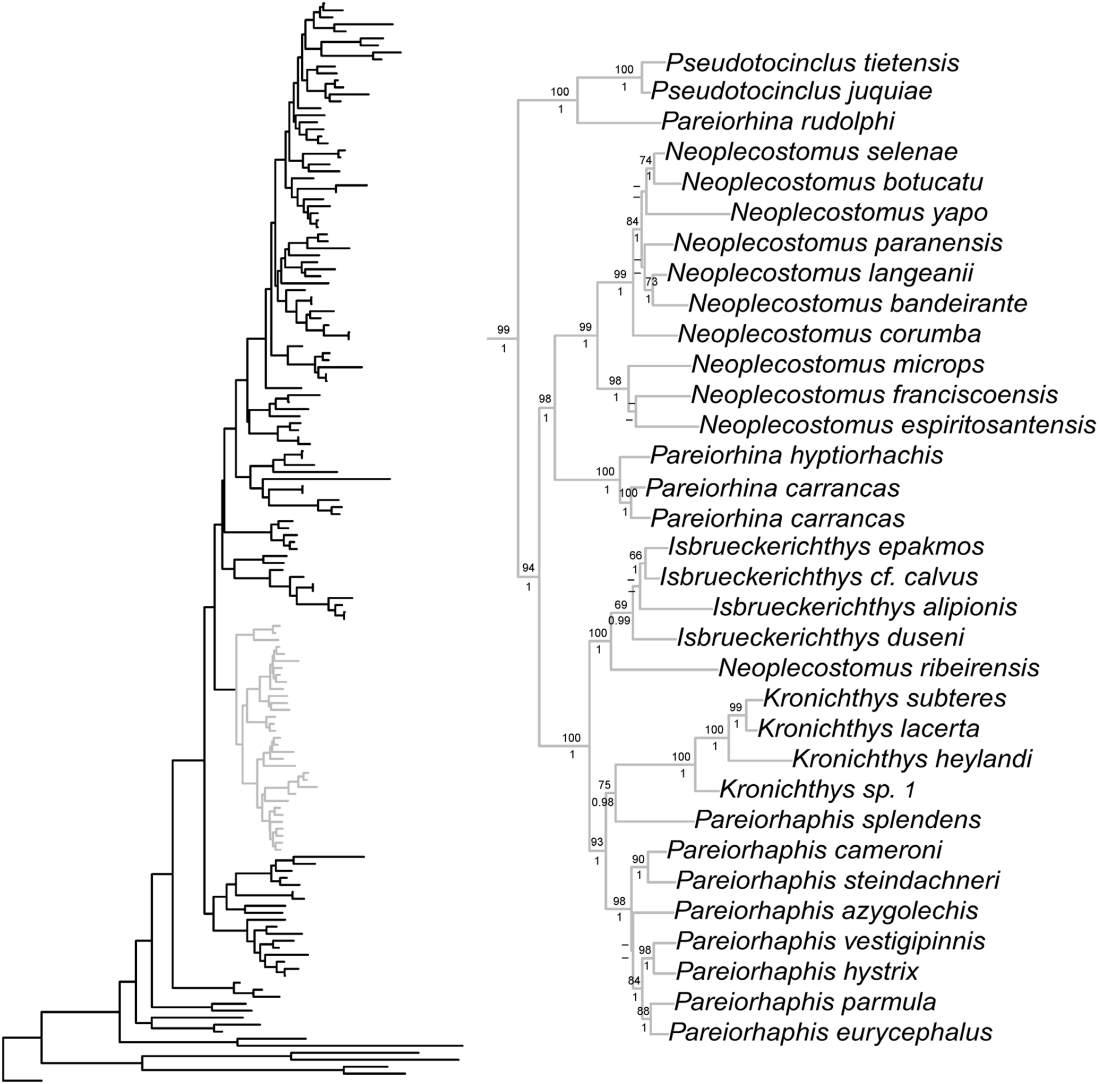
Partial ML tree showing interrelationship among species of the subfamily Neoplecostominae. Numbers above branches are bootstrap values from 1000 bootstrap pseudoreplicates obtained from ML analysis. Bootstrap values below 50% (−) are not shown. Numbers below branches are posterior probabilities obtained in the BI analysis. Posterior probabilities values below 0.95 (−) or when the nodes were not obtained by B analyses are not shown.

**Figure 3:**
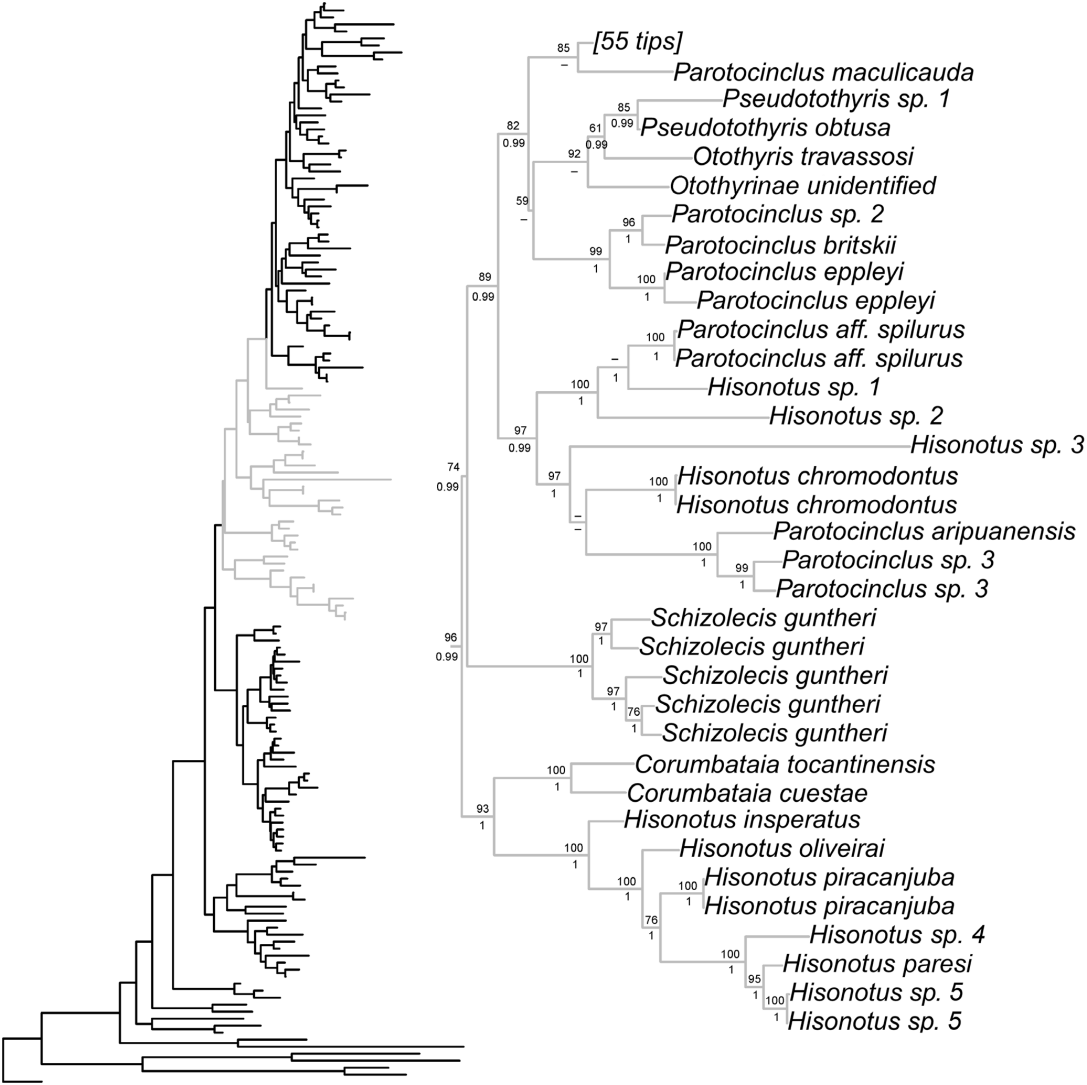
Partial ML tree showing interrelationship among species of the subfamily Otothyrinae. Numbers above branches are bootstrap values from 1000 bootstrap pseudoreplicates obtained from ML analysis. Bootstrap values below 50% (−) are not shown. Numbers below branches are posterior probabilities obtained in the BI analysis. Posterior probabilities values below 0.95 (−) or when the nodes were not obtained by B analyses are not shown.

**Figure 4:**
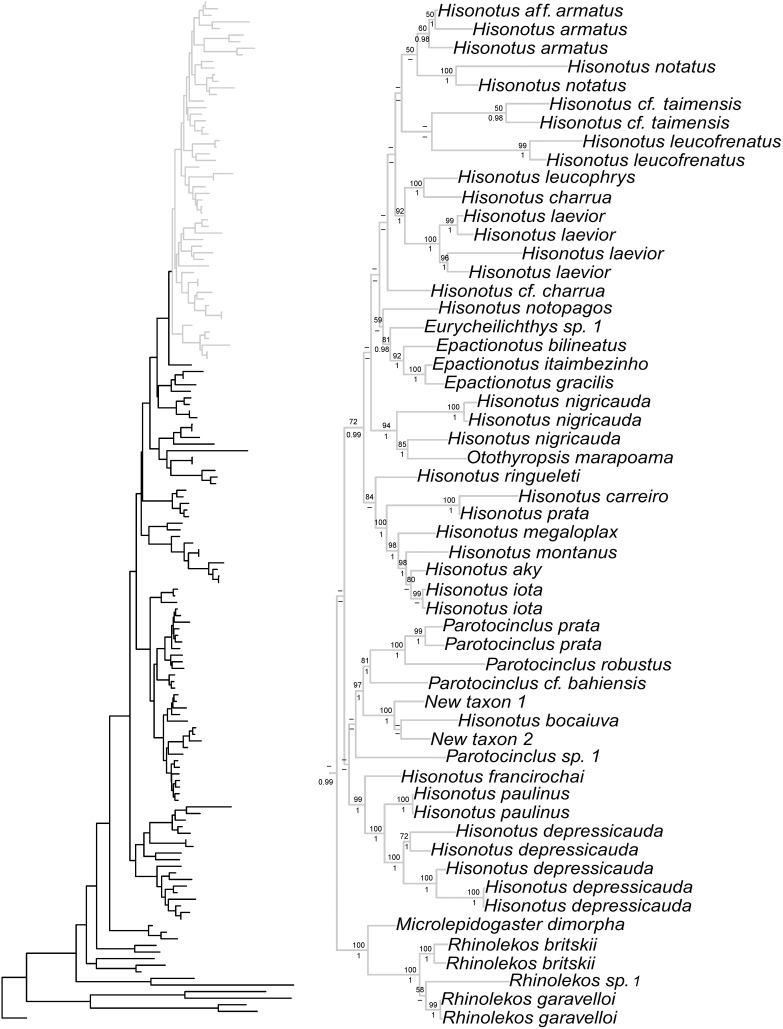
Partial ML tree showing the interrelationship among species of the subfamily Otothyrinae. Numbers above branches are bootstrap values from 1000 bootstrap pseudoreplicates obtained from ML analysis. Bootstrap values below 50% (−) are not shown. Numbers below branches are posterior probabilities obtained in the BI analysis. Posterior probabilities values below 0.95 (−) or when the nodes were not obtained by B analyses are not shown.

Bayesian and ML phylogenetic analyses resulted in very similar topologies (Figs. 1–4). Our results illustrate that the clades Hypoptopomatinae, Neoplecostominae and Otothyrinae are monophyletic with strong statistical support (BS = 96, P = 0.99 for Hypoptopomatinae; BS = 99, P = 1.00 for Neoplecostominae; BS = 96, P = 0.99 with BI for Otothyrinae). Additionally, our results suggest that Neoplecostominae is more closely related to Otothyrinae than to Hypoptopomatinae (BS = 98, P = 0.99), and that these two clades together form the sister group to Hypoptopomatinae to the exclusion of other Loricariidae (BS = 97, P = 1.00). Tree topology tests rejected the hypothesis that Otothyrinae and Hypoptopomatinae are sister groups (as proposed by Schaefer 1991 and 1998) in two (ELW and AU) of the three tests performed (Table S7). The hypothesis that Otothyrinae and Hypoptopomatinae are sister taxa was not supported by the SH test, but this test is considered less reliable than the AU test for the same datasets [76].

Within Hypoptopomatinae all examined genera were recovered as monophyletic with strong statistical support values (BS = 69, P = 1.0 for *Otocinclus*; BS = 97, P = 1.0 for *Oxyropsis*; BS = 100, P = 1 for *Acestridium*; BS = 100, P = 1 for *Hypoptopoma*). *Otocinclus* was recovered as the sister group of *Lampiella gibbosa*, and these taxa together formed the sister group to a clade consisting of *Oxyropsis*, *Acestridium* and *Hypoptopoma*. *Acestridium* and *Hypoptopoma* group together as the sister group to *Oxyropsis*.

Within Neoplecostominae *Kronichthys* and *Isbrueckerichthys* were recovered as monophyletic with high statistical support (BS = 100, P = 1.0 for *Kronichthys*; BS = 69, P = 0.99 for *Isbrueckerichthys*), however *Pareiorhaphis*, *Pareiorhina* and *Neoplecostomus* were not recovered as monophyletic. The topology tests rejected the hypothesis of a monophyletic *Neoplecostomus* and *Pareiorhina* (Table S7). *Pareiorhaphis splendens* formed the sister group to species of *Kronichthys*, and this group formed the sister taxon to other species of *Pareiorhaphis*.

Within Otothyrinae *Corumbataia*, *Schizolecis*, *Rhinolekos*, *Epactionotus* and *Pseudotothyris* were monophyletic with high statistical support (BS = 100, P = 1.0 for *Corumbataia*; BS = 100, P = 1.0 for *Schizolecis*; BS = 100, P = 1.0 with BI for *Rhinolekos*; BS = 92, P = 1.0 for *Epactionotus*, BS = 85, P = 0.99 for *Pseudotothyris*). The genera *Hisonotus* and *Parotocinclus* were not monophyletic. There are four lineages within the subfamily Otothyrinae that include species currently assigned to *Hisonotus*. The first lineage includes the species *Hisonotus insperatus*, *H. oliveirai*, *H. paresi*, *H. piracanjuba*, *Hisonotus* sp. 4, and *Hisonotus* sp. 5 and is supported by high statistical support values (BS = 100 with ML and P = 1). The second lineage is composed of the species *Hisonotus chromodontus*, *Hisonotus* sp. 1, *Hisonotus* sp. 2, *Hisonotus* sp. 3, *Parotocinclus aripuanensis*, *Parotocinclus* aff. *spilurus*, and *Parotocinclus* sp. 3. The third lineage is composed of *Hisonotus depressicauda*, *H. francirochai* and *H. paulinus*, and is supported by high statistical support values (BS = 99, P = 1.0). The fourth lineage is composed of the most number of *Hisonotus* species in this analysis, including *Hisonotus aky*, *H. iota*, *H. montanus*, *H. megaloplax*, *H. prata*, *H. carreiro*, *H. ringueleti*, *H. nigricauda*, *H. notopagos*, *H.* cf. *charrua*, *H. laevior*, *H. charrua*, *H. leucophrys*, *H. leucofrenatus*, *H.* cf. *taimensis*, *H. notatus* and *H. armatus*, and for species *Otothyropsis marapoama*, *Eurycheilichthys* sp. 1, *Epactionotus bilineatus*, *E. itaimbezinho* and *E. gracilis*, and is supported by high statistical support values (BS = 72, P = 0.99).

### Relaxed Clocks and Historical Biogeography

**Figure 5:**
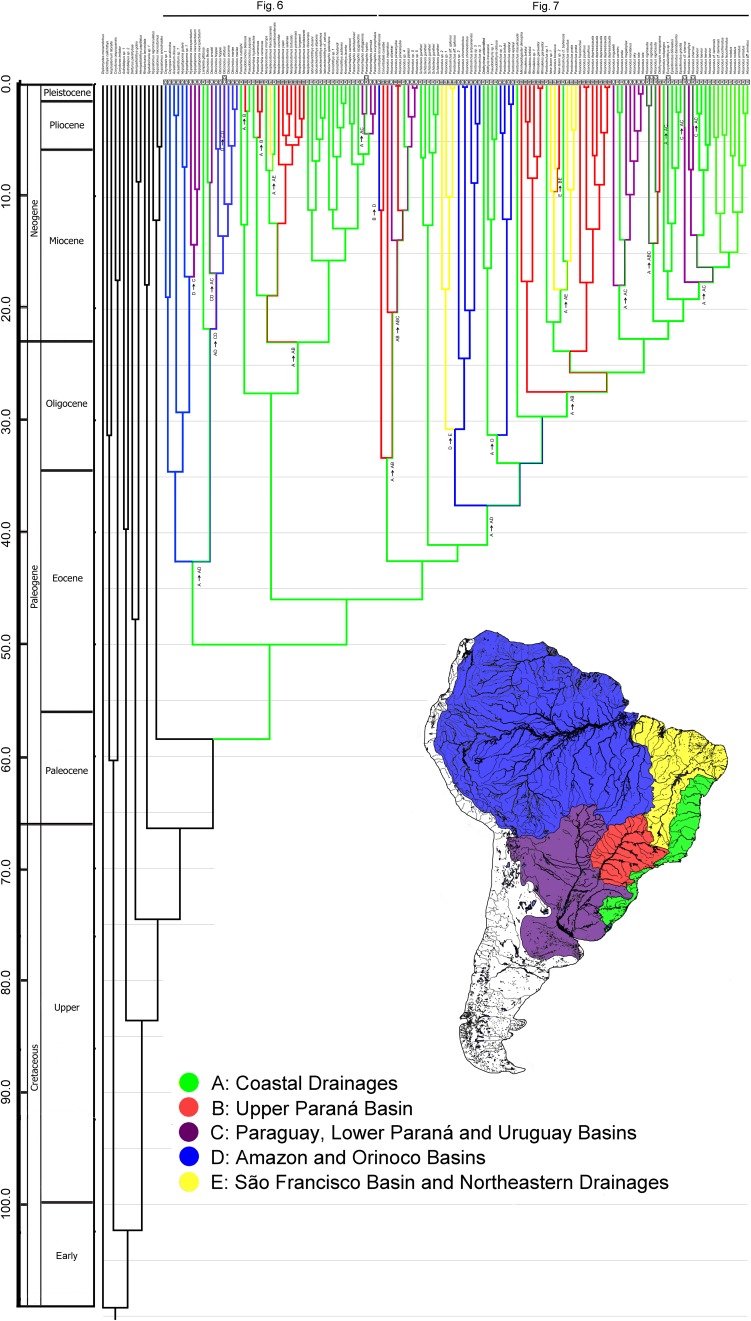
Time-calibrated phylogeny for Hypoptopomatinae, Neoplecostominae and Otothyrinae. Tree topology from BEAST analysis of 155 specimens representing 113 loricariid species. Divergence ages calibrated by origins of Siluriformes (120 Mya) and Callichthyidae (55 Mya). Regions: A, Atlantic Coastal Drainages (Green); B, Upper Paraná Basin (Red); C, Paraguay, Lower Paraná and Uruguay Basins (Purple); D, Amazon and Orinoco Basins (Blue); E, São Francisco Basin and Northeastern Drainages (Yellow).

**Figure 6:**
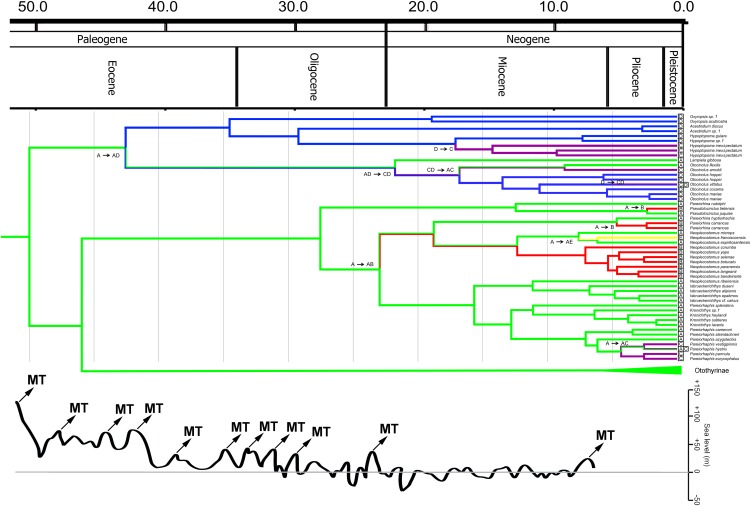
Partial time-calibrated tree from BEAST analysis, showing divergence ages for taxa in Hypoptopomatinae and Neoplecostominae. The curve below the phylogeny represented sea levels with the marine transgressions (MT), modified from Zachos et al. [124] and Miller et al. [125].

**Figure 7:**
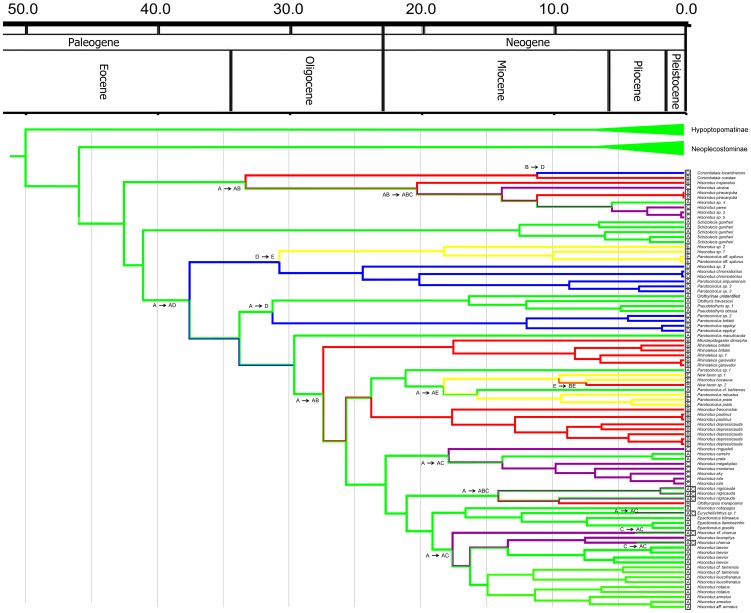
Partial time-calibrated tree from BEAST analysis, showing divergence ages for taxa in Otothyrinae.

Our time tree (Figs. 5–7) is the most comprehensive study of catfishes of Hypoptopomatinae, Neoplecostominae and Otothyrinae to date, including 115 loricariid species in these three subfamilies. The mean substitution rate for the dataset estimated using BEAST is 0.272% per MY. The Hypoptopomatinae is estimated by BEAST to have originated during the Lower Eocene about 33.6–67.4 Mya 95% HPD (mean 49.9 Mya), and is inferred by Lagrange to have originated in the Atlantic Coastal Drainages region (Fig. 5, Region A). The clade composed of Neoplecostominae + Otothyrinae is estimated by BEAST to also have originated during the Lower Eocene about 31.0–62.2 Mya 95% HPD (mean 45.9 Mya), and is also inferred by Lagrange to have originated in the Atlantic Coastal Drainages region (Fig. 5, Region A).

Hypoptopomatinae is distributed across three of the geographic regions in Fig. 5: Atlantic Coastal Drainages (Region A), Paraguay, Lower Paraná and Uruguay Basins (Region C), and Amazon and Orinoco Basins (Region D). The ancestral-area estimations suggest that the Hypoptopomatinae moved from Coastal Drainages (Region A) to the Amazon and Orinoco Basins (Region D) between 26.7–58.6 Mya 95% HPD (mean 42.5 Mya). The relationships among hypoptopomatine taxa in the Amazon and Paraguay basins also suggest that these two regions were either connected or exchanged headwaters at about 15 Mya (Fig. 6).

Neoplecostominae is also distributed across three of the regions in Fig. 5: Coastal Drainages (Region A), Upper Paraná Basin (Region B) and São Francisco Basin and Northeastern Drainages (Region E). The ancestral lineage of *Neoplecostomus* (except *N. ribeirensis*), *Pareiorhina carrancas* is inferred to have reached the Upper Paraná Basin from the Coastal Drainages at c. 14.2–33.4 Mya 95% HPD (mean 22.9 Mya). The ancestor of *Pareiorhina carrancas* reached the Upper Paraná Basin from the Coastal Drainages at c. 1.7–8.7 Mya 95% HPD (mean 4.6 Mya). The ancestor of *Neoplecostomus franciscoensis* reached the São Francisco basin from Coastal Drainages at c. 3.9–13.1 Mya 95% HPD (mean 7.5 Mya). The ancestor of *Pseudotocinclus tietensis* reached the Upper Paraná Basin (B) from the Coastal Drainages region (A) about 0.4–5.5 Mya 95% HPD (mean 2.3 Mya). The ancestral lineage of *Pareiorhaphis eurycephalus*, *P. hystrix*, *P. parmula* and *P. vestigipinnis* reached the Uruguay Basins about 2.0–7.5 Mya 95% HPD (mean 4.3 Mya) (Fig. 6).

The ancestral-area estimations (Fig. 7) suggest that Otothyrinae originated in the Atlantic Coastal Drainages (Region A) and then subsequently expanded its range into the other regions by means of biotic dispersal, geodispersal (river capture), or both. The first group to diverge within Otothyrinae is composed for species of the genus *Corumbataia* and six species of the genus *Hisonotus* (*H. insperatus*, *Hisonotus oliveirai*, *Hisonotus paresi*, *H. piracanjuba*, *Hisonotus* sp. 4 and *Hisonotus* sp. 5). The ancestral lineage of this group originated in Coastal Drainages region (A) at 29.0–57.1 Mya 95% HPD (mean 42.5 Mya). The second group to diverge is composed of *Schizolecis guntheri,* the only known species of *Schizolecis*. Our results suggest that the ancestor of this species originated in the Coastal Drainages region (A) about 28.7–55.8 Mya 95% HPD (mean 41.0 Mya). The third group to diverge within Otothyrinae is composed four species of *Hisonotus* (*Hisonotus* sp. 1, *Hisonotus* sp. 2, *Hisonotus* sp. 3 and *Hisonotus chromodontus*) and three species of *Parotocinclus* (*Parotocinclus* sp. 3, *P. aripuanensis* and *Parotocinclus* aff. *spilurus*). The ancestor of this group dispersed from the Coastal Drainages region (A) to the Amazon and Orinoco Basins (D) about 25.6–51.0 Mya 95% HPD (mean 37.5 Mya). Subsequently, the ancestor of the clade composed of *Hisonotus* sp. 1, *Hisonotus* sp. 2 and *Parotocinclus* aff. *spilurus* reached the São Francisco Basin and Northeastern Basins (E) about 19.3–43.2 Mya 95% HPD (mean 30.7 Mya).

The fourth group to diverge within Otothyrinae is composed of species of the genus *Pseudotothyris*, *Otothyris*, a Otothyrinae unidentified and the species *Parotocinclus* sp. 2, *P. britskii* and *P. eppleyi*. The ancestor of this group originated in the Coastal Drainages region (A) about 23.3–46.1 Mya 95% HPD (mean 33.7 Mya). Subsequently, the ancestor of *Parotocinclus* sp. 2, *P. britskii* and *P. eppleyi* dispersed from to the Amazon and Orinoco Basins (D) about 19.8–42.6 Mya 95% HPD (mean 31.2 Mya). The ancestor of the group composed of the species *Microlepidogaster dimorpha*, *Rhinolekos* sp. 1, *R. britskii* and *R. garavelloi* dispersed from the Coastal Drainages region (A) to the Upper Paraná Basin (B) about 18.3–37.3 Mya 95% HPD (mean 27.3 Mya). The ancestor of the clade composed of *Hisonotus depressicauda*, *H. francirochai* and *H. paulinus* originated in Upper Paraná Basin (B) about 15.7–33.2 95% HPD (mean 23.7 Mya). The ancestor of the clade composed of *Parotocinclus* sp. 1, *Parotocinclus* cf. *bahiensis*, *P. robustus* and *P. prata*, New taxon sp. 1, New taxon sp. 2 and *Hisonotus bocaiuva*, originated in the Coastal Drainages region (A) about 15.7–33.2 Mya 95% HPD (mean 23.7 Mya). The ancestral of the clade composed of the most species of *Hisonotus* and it type species *H. notatus* its closest relatives (*Hisonotus aky*, *H. iota*, *H. montanus*, *H. megaloplax*, *H. prata*, *H. carreiro*, *H. ringueleti*, *H. nigricauda*, *H. notopagos*, *H.* cf. *charrua*, *H. laevior*, *H. charrua*, *H. leucophrys*, *H. leucofrenatus*, *H.* cf. *taimensis*, *H. notatus* and *H. armatus*), and the species *Otothyropsis marapoama*, *Eurycheilichthys* sp. 1, *Epactionotus bilineatus*, *E. itaimbezinho* and *E. gracilis* originated in the Coastal Drainage region about 17.0–35.0 Mya 95% HPD (mean 25.6 Mya).

Additionally, two important dispersal events can be inferred from the Coastal Drainages region (A) to the Paraguay, Lower Paraná and Uruguay Basins (C). The first is the ancestor of *Hisonotus iota*, *H. aky*, *H. montanus*, *H. megaloplax*, *H. prata*, *H. carreiro* and *H. ringueleti* about 10.7–26.3 Mya 95% HPD (mean 17.8 Mya). The second is the ancestor of *Hisonotus cf. charrua*, *H. leucophrys*, *H. charrua*, *H. laevior*, *H.* cf. *taimensis*, *H. leucofrenatus*, *H. notatus* and *H. armatus* about 11.4–24.8 95% HPD (mean 17.5 Mya).

## Discussion

### River capture as a biogeographic process

In this study we used a time-calibrated molecular phylogeny and ancestral-area estimations (Figs. 5–7) with robust taxonomic sampling, to document the effects of river capture on the diversification of taxa in the HNO-clade of loricariid (armoured) catfishes in South America. The results are largely consistent with those of previous studies of loricariids from Southern and Southeastern Brazil [36], [48], [85]. For example, Chiachio et al. [36] recovered a similar division of the HNO-clade into two monophyletic groups, the Hypoptopomatinae and Neoplecostominae + Otothyrinae, inferred the ancestor of Hypoptopomatinae to have inhabited the Amazon basin, and inferred the ancestor of Neoplecostominae + Otothyrinae to have inhabited an area now drained by the Upper Parana and part of the Atlantic coastal drainages.

The Atlantic coastal region has a complex and ancient geological history that traces to the final separation of Africa and South America about 100 million years ago [86]–[89]. Roxo et al. [48] identified the Coastal Drainages of Southeastern Brazil as an important area where many lineages of Loricariidae originated, including the ancestors of Neoplecostominae. Ribeiro [16] described a set of phylogenetic patterns (termed A, B and C) with sister group relationships observed between lineages inhabiting the Atlantic coastal drainages and inland drainages such as Amazon and Paraná Basins. Our results suggest a fit to pattern B in Neoplecostominae and Otothyrinae, with sister-group relationships between species endemic to the Brazilian coastal drainages and adjacent portions of the Brazilian shield. Ribeiro [16] listed *Lignobrycon*, *Rhinelepis*, *Spintherobolus*, and *Triportheus*, the tribes Aspidoradini and Glandulocaudini, and the subfamilies Cheirodontinae and Sarcoglanidinae as examples of pattern B.

According to the Lagrange ancestral-area estimations, the area of the modern Atlantic Coastal Drainages (Region A) is optimized as the ancestral area for three of the deepest nodes of the HNO phylogeny. These nodes include the HNO-clade as a whole (40.8–79.7 Mya 95% HPD, mean 58.4 Mya, Fig. 5), the Hypoptopomatinae (33.6–67.4 Mya 95% HPD, mean 49.9 Mya, Fig. 6), and the Neoplecostominae and Otothyrinae (31.0–62.2 Mya 95% HPD, mean 45.9 Mya, Fig. 6–7). The results of the Lagrange analysis are consistent with a river capture event at about 26.7–58.6 Mya 95% HPD (mean 42.5 Mya), allowing range expansion(s) from the Atlantic Coastal Drainages (Region A) to a region comprised of the modern Paraguay/Lower Paraná/Uruguay (Region C) and Amazon/Orinoco Basins (Region D). An important river capture event at this approximate time and place is also consistent with the topology of a General Area Cladogram of fish taxa from tropical South America, as inferred from a Brooks Parsimony (meta) Analysis of all 32 published phylogenies of species-rich fish clades available at that time [22].

Chiachio et al. [36] explained the division of Hypoptopomatinae, between lineages in the Amazon Basin (Region D) and the Brazilian East Coastal (Region A) and the Upper Paraná (Region B), as the result of limited dispersal of fishes to less favourable areas of the continental margin. Although species of Hypoptopomatinae do inhabit lowland rivers in the Amazon, Orinoco and Guianas regions, most species of Neoplecostominae plus Otothyrinae inhabit rivers and streams in the mountainous Brazilian Shield, where they are adapted to colder and more highly oxygenated waters [27]. Additionally, historical paleogeographic connections among the Orinoco, Amazon, and Paraguay basins are hypothesized to have enabled the colonization of Hypoptopomatinae species through these basins [4].

Within Neoplecostominae our time-calibrated phylogeny and Lagrange biogeographic analysis suggest a geodispersal event in the ancestral species of the clade composed of *Neoplecostomus* (except *N. ribeirensis*) and *Pareiorhina carrancas* to move from the Coastal Drainages (Region A) to the Upper Paraná Basin (Region B) at about 14.2–33.4 Mya 95% HPD (mean 22.9 Mya). Roxo et al. [48] reported an event with a similar date in the range 15.4–38.1 Mya 95% HPD (mean 26.7 Mya), and suggested that this geodispersal event could be a result of a headwater capture. During this time period several headwater capture events have been proposed between the Rio Tietê, Rio Paraíba do Sul, Rio São Francisco, and Rio Ribeira de Iguape basins [16], [90], [91]. Headwater capture is likely to have influenced ancestral fish distributions throughout adjacent drainages, allowing the ancestors of this group to reach the Upper Paraná basin.

The subfamily Otothyrinae also has a complex biogeographic history among South American basins (Fig. 7). The ancestral-area estimation with highest ML scores gives us the origin in the Coastal Drainages (Region A). The first lineage to diverge within Otothyrinae (i.e. species of *Corumbataia* and six species of *Hisonotus, H. insperatus*, *H. oliveirai*, *H. paresi*, *H. piracanjuba, Hisonotus* sp. 4 and *Hisonotus* sp. 5), geodispersal from Coastal Drainage (Region A) to Upper Paraná basin (Region B) and is estimated in the time frame 20.7–47.4 Mya 95% HPD (mean 33.29 Mya).

The results of our Lagrange analysis suggest the influence of river capture in the movement of Otothyrinae from Atlantic Coastal Drainages (Region A) to the Amazon and Orinoco Basins (Region D). The most highly supported model (M3) of geographic dispersal among areas posits a connection between regions C (Paraguay, Lower Paraná and Uruguay Basins) and D (Amazon and Orinoco Basins) before 15 Mya. For more than a century authors have suggested historical dispersal routs of fishes between Paraguay and Amazon basins [20], [25], [92]–[95]. These authors suggested that most of the fish lineages represented in the Paraguay Basin can be explained by dispersal, presumably by means of headwater capture (geodispersal) of Amazon tributaries (Madeira, Tocantins, Xingu) on the Brazilian Shield. However, geodispersal events in the reverse direction, from south to north, must also be considered for taxa with origins in the La Plata and Atlantic coastal drainages, and with derived lineages in the Amazon and Orinoco basins.

The Lagrange analysis also infers a river capture event affecting the ancestor of the clade including New taxon sp. 1, New taxon sp. 2, *Hisonotus bocaiuva*, *Parotocinclus* cf. *bahiensis*, *P. robustus* and *P. prata* from the Atlantic Coastal Drainages (Region A) to the São Francisco Basin and Northeastern Drainages (Region E) in the time frame 11.3–26.1 Mya 95% HPD (mean 18.2 Mya). These two regions also share extensive watershed divides with the many separate Atlantic coastal drainages of the eastern margin of the Brazilian Shield. Ribeiro [16] suggested that the origin of the Taubaté Graben probably resulted in the capture of several other adjacent rivers, such as headwaters of the Tietê, Grande, São Francisco and Doce rivers. A river capture event at this approximate time and place is also consistent with the General Area Cladogram of fish taxa from tropical South America [22].

The results of our Lagrange analysis point to the influence of several river-capture events permitting movements of Otothyrinae lineages from the Atlantic Coastal Drainages (Region A) to the Paraguay, Lower Paraná and Uruguay Basins (Region C; Fig. 7). These events occurred within the group of *Hisonotus* species (including the type species *Hisonotus notatus*), and the species of the genera *Eurycheilichthys* and *Epactionotus*. While most of the early-branching clades in this group inhabit the eastern margin of the Brazilian Shield, a few early-branching lineages occur in the Uruguay Basin (Region C). Ribeiro [16] reported that several species are shared between the isolated coastal drainages and the adjacent upland as: *Cnesterodon decemmaculatus* and *Cnesterodon brevirostratus* [96], *Bryconamericus patriciae* [97], *Hypostomus commersoni* and *H. aspilogaster* [98].

The results of our Lagrange analysis also indicate a geodispersal event from the Amazon and Orinoco basins (Region D) to the São Francisco Basin and Northeastern Drainages (Region E) in the ancestor of *Hisonotus* sp. 1, *Hisonotus* sp. 2 and *Parotocinclus* aff. *spilurus* at about 19.3–43.2 Mya 95% HPD (mean 30.7 Mya). Rosa et al. [99] previously suggested that some fish species in Northeastern Brazil are widespread in two or more basins, encompassing the São Francisco, Parnaíba and several adjacent coastal rivers basins. This is the case, for example, in *Triportheus signatus, Prochilodus brevis*, *Cichlasoma orientale* and *Parauchenipterus galeatus*.

#### 4.2. Sea-level changes as a biogeographic process

Periods of alternating sea-level stands can also influence the distributions of lowland freshwater taxa [100]–[102]. Eustatic sea-level changes under global climate controls, and regional subsidence or uplift under tectonic controls, have resulted in multiple marine transgressions and regressions over the course of the Cenozoic, alternately flooding and exposing low lying areas of the continental platforms, and converting lowland and coastal plains from freshwater to shallow marine ecosystems. López-Fernandez and Albert [103] identify six marine transgressions during the Eocene, one in the Eocene and Oligocene, three in Oligocene and one in the Miocene, the time interval during which most lineages of Hypoptopomatinae diversified (Fig. 6). Ancestral lineages of Hypoptopomatinae were present in the lowland portions of the Amazon and Orinoco basins (Region D) from about 26.7–58.6 Mya 95% HPD (mean 42.5 Mya) to the present, and our results suggests three events of geodispersal to the lowland portions of the La Plata basin from about 12.6–33.1 Mya 95% HPD (mean 21.7 Mya), 9.9–26.4 Mya 95% HPD (mean 17.0 Mya) and 0.0–10.6 Mya 95% HPD (mean 5.3 Mya) to the present. These populations were therefore presumably influenced by numerous regional marine transgressions and regressions.

Marine transgressions can isolate and fragment lowland fish populations, promoting both speciation and extinction by reducing the total amount and connectivity of freshwater habitat patches [11], [16], [100], [103], [104]. Marine transgressions can also result in local population extirpations and/or allopatric speciation in upland refugia (e.g. Albert et al. [105]. Marine regressions can expand lowland and coastal freshwater habitats, thereby promoting dispersal and reducing extinction [103], [106].

The relatively small areal extent of river basins in the Atlantic coastal drainages, combined with areal expansions and contractions due to Pleistocene shoreline fluctuations, may have acted in concert to elevate speciation and extinction rates in this region [107], [108]. Indeed many extant fish species in the Atlantic coastal drainages are of high conservation concern [109]. However, the effect of Pleistocene shoreline fluctuations on fish diversity was presumably restricted to the coastal plain (areas below 100m elevation), whereas most of the fish species of the Atlantic coastal drainages inhabit canyons in the piedmont, especially larger rivers such as the Rio Doce and Ribeiro de Iguape [110].

### Peripheral location of low-diversity, early-branching lineages

The ancestral-area estimations generated by Lagrange (Fig. 5) permit one to infer the geographic origin of the HNO-clade, and of all three HNO subfamilies, in the Atlantic Coastal Drainages (Region A), a relatively narrow strip of rivers basins that extends along the eastern continental margin. This is a reasonable interpretation given the disproportionately high number of low-diversity, early-branching clades in all three subfamilies restricted to Region A. This interpretation also conforms to widespread expectations about the relative rates of macroevolutionary parameters that affect net rates of diversification [111]–[113]. The Lagrange DEC model of species range evolution assumes a model of biogeographic history dominated by vicariance, in which dispersal and extinction are treated as relatively rare events [84], [114]. The Lagrange model is also entirely neutral (*sensu* Hubbell [115]) with respect to DEC parameter values among clades and regions.

An alternative interpretation of HNO biogeographic history may also be considered, in which the ancestral species range was distributed over a wide portion of southern South America in the early Cenozoic, including much of the modern Atlantic Coastal (Region A), Upper Parana (Region B), and Paraguay/Lower Parana/Uruguay (Region C) areas. Under this alternative interpretation, the accumulation of many low-diversity, early-branching clades in the Atlantic coastal drainages is expected from patterns of diversification on landscapes with low rates of river capture.

In places like the eastern margin of South America, where geographic range evolution is thought to have been dominated by river capture [16], [116]–[118], vicariance and geodispersal events are expected to be coupled (see the Introduction; see also Albert and Crampton [7]). Under these conditions, rates of speciation and dispersal should be approximately matched as sources for the introduction of new species (*sensu* Vellend [119]). Further, because dispersal expands species ranges, it tends to reduce extinction rates, and freshwater fish species with larger ranges generally have lower extinction risk [120], [121]. Therefore, the combination of low speciation and extinction rates in the Atlantic coastal drainages may have contributed to an accumulation of low-diversity clades. By contrast, the relatively higher rates of speciation and extinction in the La Plata basin is predicted to have resulted in a phylogeny with few or no low-diversity early-branching clades (see e.g. Albert et al. [4] fig. 2.15). In other words, regions with high species turnover are less likely to retain low-diversity early-branching clades (i.e. the Effect Hypothesis of Vrba [122]).

This alternative interpretation predicts the presence of many low-diversity, early-branching fish lineages on landscapes with low rates of river capture. This alternative interpretation differs from the Lagrange-generated ancestral-area estimations by positing different rates of speciation and extinction in clades inhabiting the Atlantic coastal drainages and La Plata basin. In other words, this alternative interpretation is not neutral with respect to DEC parameter values among regions, positing instead that rates of speciation and extinction are correlated with rates of river capture.

### Museums and cradles

In evaluating distributional patterns of Neotropical fish distributions in southeastern Brazil, Ribeiro [16] concluded that the Atlantic coastal drainages (Region A of the present study) served as both a cradle and a museum of diversity for different fish groups. The terms “evolutionary cradle” and “evolutionary museum” are alternative hypotheses for the occurrence of areas with high species richness [123]. An “evolutionary cradle” is an area with high rates of speciation, where environmental conditions promote speciation. By contrast, an “evolutionary museum” is an area with low rates of extinction, where low rates of environmental disturbance act to preserve early-branching taxa, and where species richness accumulates through long periods of geological time.

**Figure 8:**
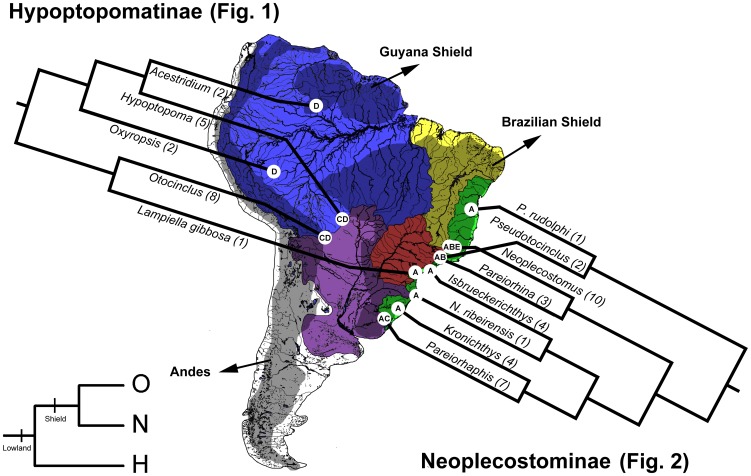
Geographic distribution and phylogeny of major clades of Hypoptopomatinae and Neoplecostominae in tropical South America. Terminal taxa may represent species or monophyletic higher taxa (see figs. 1–2 for all taxa). Numbers in parentheses after each taxon name are number of taxa sampled for each lineage. Regions: A, Coastal Drainages (Green); B, Upper Paraná Basin (Red); C, Paraguay, Lower Paraná and Uruguay Basins (Purple); D, Amazon and Orinoco Basins (Blue); E, São Francisco Basin and Northeastern Drainages (Yellow).

**Figure 9:**
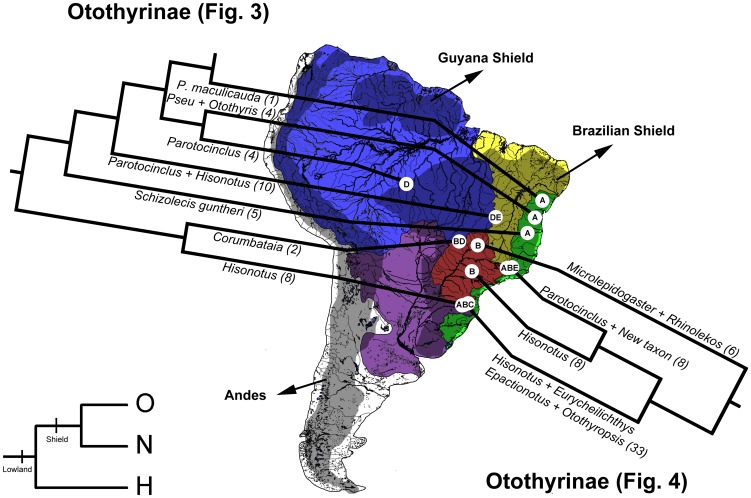
Geographic distribution and phylogeny of major clades of Otothyrinae in tropical South America. Terminal taxa may represent species or monophyletic higher taxa (see figs. 1–2 for all taxa). Numbers in parentheses after each taxon name are number of taxa sampled for each lineage. Regions: A, Coastal Drainages (Green); B, Upper Paraná Basin (Red); C, Paraguay, Lower Paraná and Uruguay Basins (Purple); D, Amazon and Orinoco Basins (Blue); E, São Francisco Basin and Northeastern Drainages (Yellow).

The results of this study on the Hypoptopomatinae, Neoplecostominae and Otothyrinae broadly concur with the conclusions of Ribeiro [16] (Figs. 8–9). All three HNO subfamilies are inferred by Lagrange ancestral-area estimations to have originated in the Atlantic coastal drainages, suggesting that this region served as the cradle for early diversification in these clades. In addition, several lineages of Neoplecostominae remain confined to the region of the Atlantic coastal drainages, which therefore also appears to serve as a museum for these clades. These major patterns of diversification in Neoplecostominae in the Atlantic coastal drainages and Brazilian Shield were previously recognized by Roxo et al. [48]. For Hypoptopomatinae, most of the diversification occurred in lowlands of the Amazon, Orinoco and Paraguay basins, and the species *Lampiella gibbosa* and *Otocinclus affinis* appears to be relictual lineages confined to the Atlantic coastal drainages. Diversification within Otothyrinae exhibits a pattern with monophyletic lineages in each of the several regions and basins of the South American platform (Fig. 9).

Here, we presented a time-calibrated phylogenetic analysis of the loricariid catfish subfamilies Hypoptopomatinae, Neoplecostominae and Otothyrinae, used parametric biogeographic methods to estimate ancestral geographic ranges, and documented several historical river-capture events. The results of this study largely support previous hypotheses on the origins and evolution of the major HNO-clades in space and time. The historical biogeographic analysis of these taxa also illustrates the role of river capture as an important evolutionary process contributing to the extraordinary diversification of Neotropical fishes in Southeastern Brazil.

## Supporting Information

Table S1 Species included in the present study.
(DOCX)

Table S2 Summary of taxonomic information for species of Hypoptopomatinae, Neoplecostominae and Otothyrinae included in the analysis.
(DOC)

Table S3 Primers used in the present study to amplify partial sequences of F-reticulon 4, 16S rRNA, cytochrome oxidase subunit I (COI) and cytochrome B (CytB).
(DOC)

Table S4 Nucleotide substitution models for each partition evaluated in the software PartitionFinder [68] and used in the phylogenetic analyses. *These partitions were analyzed in one partition.
(DOC)

Table S5 DEC models tested to estimate distribution ranges inherited by the descending lineages at each node of the tree. The differences between the models are in the rate of dispersal among adjacent and no adjacent areas. * Represent the model used in the analysis.
(DOC)

Table S6 Substitution Saturation estimated for each gene using the index of substitution saturation (Iss) [65], [66] and the rate of transitions/transversions evaluated in software DAMBE 5.2.31 [67].
(DOC)

Table S7 Likelihood-based tests for alternative topologies. SH and AU are probability values obtained for the Shimodaira-Hasegawa and the Approximately Unbiased tests [76]. Asterisks denote significant values (P<0.05 for SH and P<0.01 for AU and ELW), that imply the topology is rejected.
(DOC)
