# Supplementary material for: Molecular Phylogeny and Biogeographic History of the Armored Neotropical Catfish Subfamilies Hypoptopomatinae, Neoplecostominae and Otothyrinae (Siluriformes: Loricariidae)
Source: PLoS One. 2014 Aug 22;9(8):e105564. doi: 10.1371/journal.pone.0105564 (PMC4141799; doi:10.1371/journal.pone.0105564)
Supplement: Table S2 — Summary of taxonomic information for species of Hypoptopomatinae, Neoplecostominae and Otothyrinae included in the analysis. (DOC) [file pone.0105564.s002.doc]

**Table 2.** Summary of taxonomic information for species of Hypoptopomatinae, Neoplecostominae and Otothyrinae included in the analysis. Total number of valid names from Eschmeyer and Fong [39].

| **Subfamily** | **Genus** | **Species** | **Areas** | | |
| --- | --- | --- | --- | --- | --- |
| **Otothyrinae** | *Corumbataia* | *cuestae* | B | | |
| **Otothyrinae** | *Corumbataia* | *tocantinensis* | D | | |
| **Otothyrinae** | *Eurycheilichthys* | sp. 1 | A/C | | |
| **Otothyrinae** | *Epactionotus* | *bilineatus* | A | | |
| **Otothyrinae** | *Epactionotus* | *gracilis* | A | | |
| **Otothyrinae** | *Epactionotus* | *itaimbezinho* | A | | |
| **Otothyrinae** | *Hisonotus* | *aky* | C | | |
| **Otothyrinae** | *Hisonotus* | *armatus* | A | | |
| **Otothyrinae** | *Hisonotus* | *armatus* | A | | |
| **Otothyrinae** | *Hisonotus* | *armatus* | A | | |
| **Otothyrinae** | *Hisonotus* | *bocaiuva* | E | | |
| **Otothyrinae** | *Hisonotus* | *carreiro* | A | | |
| **Otothyrinae** | *Hisonotus* | *charrua* | A/C | | |
| **Otothyrinae** | *Hisonotus* | cf. *charrua* | A/C | | |
| **Otothyrinae** | *Hisonotus* | *chromodontus* | D | | |
| **Otothyrinae** | *Hisonotus* | *chromodontus* | D | | |
| **Otothyrinae** | *Hisonotus* | *depressicauda* | B | | |
| **Otothyrinae** | *Hisonotus* | *depressicauda* | B | | |
| **Otothyrinae** | *Hisonotus* | *depressicauda* | B | | |
| **Otothyrinae** | *Hisonotus* | *depressicauda* | B | | |
| **Otothyrinae** | *Hisonotus* | *depressicauda* | B | | |
| **Otothyrinae** | *Hisonotus* | *francirochai* | B | | |
| **Otothyrinae** | *Hisonotus* | *heterogaster* | A | | |
| **Otothyrinae** | *Hisonotus* | *heterogaster* | A | | |
| **Otothyrinae** | *Hisonotus* | *insperatus* | B | | |
| **Otothyrinae** | *Hisonotus* | *Iota* | C | | |
| **Otothyrinae** | *Hisonotus* | *Iota* | C | | |
| **Otothyrinae** | *Hisonotus* | *laevior* | A | | |
| **Otothyrinae** | *Hisonotus* | *laevior* | A | | |
| **Otothyrinae** | *Hisonotus* | *laevior* | A | | |
| **Otothyrinae** | *Hisonotus* | *laevior* | A | | |
| **Otothyrinae** | *Hisonotus* | *leucofrenatus* | A | | |
| **Otothyrinae** | *Hisonotus* | *leucofrenatus* | A | | |
| **Otothyrinae** | *Hisonotus* | *leucophrys* | C | | |
| **Otothyrinae** | *Hisonotus* | *megaloplax* | C | | |
| **Otothyrinae** | *Hisonotus* | *montanus* | C | | |
| **Otothyrinae** | *Hisonotus* | *nigricauda* | A/C | | |
| **Otothyrinae** | *Hisonotus* | *notatus* | A | | |
| **Otothyrinae** | *Hisonotus* | *notatus* | A | | |
| **Otothyrinae** | *Hisonotus* | *notopagos* | A | | |
| **Otothyrinae** | *Hisonotus* | *oliveirai* | C | | |
| **Otothyrinae** | *Hisonotus* | *paresi* | C | | |
| **Otothyrinae** | *Hisonotus* | *paulinus* | B | | |
| **Otothyrinae** | *Hisonotus* | *paulinus* | B | | |
| **Otothyrinae** | *Hisonotus* | *piracanjuba* | B | | |
| **Otothyrinae** | *Hisonotus* | *piracanjuba* | B | | |
| **Otothyrinae** | *Hisonotus* | *prata* | A | | |
| **Otothyrinae** | *Hisonotus* | *ringueleti* | C | | |
| **Otothyrinae** | *Hisonotus* | *taimensis* | A | | |
| **Otothyrinae** | *Hisonotus* | *taimensis* | A | | |
| **Otothyrinae** | *Hisonotus* | sp. 1 | E | | |
| **Otothyrinae** | *Hisonotus* | sp. 2 | E | | |
| **Otothyrinae** | *Hisonotus* | sp. 3 | D | | |
| **Otothyrinae** | *Hisonotus* | sp. 4 | A | | |
| **Otothyrinae** | *Hisonotus* | sp. 5 | C | | |
| **Otothyrinae** | *Hisonotus* | sp. 5 | C | | |
| **Otothyrinae** | *Microlepidogaster* | *dimorpha* | B | | |
| **Otothyrinae** | *New taxon* | sp. 1 | E | | |
| **Otothyrinae** | *New taxon* | sp. 2 | B | | |
| **Otothyrinae** | *Otothyris* | *travassosi* | A | | |
| **Otothyrinae** | *Otothyropsis* | *marapoama* | B | | |
| **Otothyrinae** | *Parotocinclus* | *aripuanensis* | D | | |
| **Otothyrinae** | *Parotocinclus* | cf. *bahiensis* | A | | |
| **Otothyrinae** | *Parotocinclus* | *britskii* | D | | |
| **Otothyrinae** | *Parotocinclus* | *eppleyi* | D | | |
| **Otothyrinae** | *Parotocinclus* | *eppleyi* | D | | |
| **Otothyrinae** | *Parotocinclus* | *maculicauda* | A | | |
| **Otothyrinae** | *Parotocinclus* | *prata* | E | | |
| **Otothyrinae** | *Parotocinclus* | *prata* | E | | |
| **Otothyrinae** | *Parotocinclus* | *robustus* | E | | |
| **Otothyrinae** | *Parotocinclus* | aff. *spilurus* | E | | |
| **Otothyrinae** | *Parotocinclus* | aff. *spilurus* | E | | |
| **Otothyrinae** | *Parotocinclus* | sp. 1 | D | | |
| **Otothyrinae** | *Parotocinclus* | sp. 2 | D | | |
| **Otothyrinae** | *Parotocinclus* | sp. 3 | D | | |
| **Otothyrinae** | *Parotocinclus* | sp. 3 | D | | |
| **Otothyrinae** | *Pseudotothyris* | *obtusa* | A | | |
| **Otothyrinae** | *Pseudotothyris* | *janeirensis* | A | | |
| **Otothyrinae** | *Pseudotothyris* | sp. 1 | A | | |
| **Otothyrinae** | *Rhinolekos* | *britskii* | B | | |
| **Otothyrinae** | *Rhinolekos* | *britskii* | B | | |
| **Otothyrinae** | *Rhinolekos* | *garavelloi* | B | | |
| **Otothyrinae** | *Rhinolekos* | *garavelloi* | B | | |
| **Otothyrinae** | *Rhinolekos* | sp. 1 | B | | |
| **Otothyrinae** | *Schizolecis* | *guntheri* | A | | |
| **Otothyrinae** | *Schizolecis* | *guntheri* | A | | |
| **Otothyrinae** | *Schizolecis* | *guntheri* | A | | |
| **Otothyrinae** | *Schizolecis* | *guntheri* | A | | |
| **Otothyrinae** | *Schizolecis* | *guntheri* | A | | |
| **Neoplecostominae** | *Isbrueckerichthys* | *alipionis* | A | | |
| **Neoplecostominae** | *Isbrueckerichthys* | cf. *calvus* | A | | |
| **Neoplecostominae** | *Isbrueckerichthys* | *epakmos* | A | | |
| **Neoplecostominae** | *Isbrueckerichthys* | *duseni* | A | | |
| **Neoplecostominae** | *Kronichthys* | *heylandi* | A | | |
| **Neoplecostominae** | *Kronichthys* | *lacerta* | A | | |
| **Neoplecostominae** | *Kronichthys* | *subteres* | A | | |
| **Neoplecostominae** | *Kronichthys* | sp. 1 | A | | |
| **Neoplecostominae** | *Neoplecostomus* | *bandeirante* | B | | |
| **Neoplecostominae** | *Neoplecostomus* | *botucatu* | B | | |
| **Neoplecostominae** | *Neoplecostomus* | *corumba* | B | | |
| **Neoplecostominae** | *Neoplecostomus* | *espiritosantensis* | A | | |
| **Neoplecostominae** | *Neoplecostomus* | *franciscoensis* | E | | |
| **Neoplecostominae** | *Neoplecostomus* | *langeanii* | B | | |
| **Neoplecostominae** | *Neoplecostomus* | *microps* | A | | |
| **Neoplecostominae** | *Neoplecostomus* | *paranensis* | B | | |
| **Neoplecostominae** | *Neoplecostomus* | *ribeirensis* | A | | |
| **Neoplecostominae** | *Neoplecostomus* | *selenae* | B | | |
| **Neoplecostominae** | *Neoplecostomus* | *yapo* | B | | |
| **Neoplecostominae** | *Pareiorhaphis* | *azygolechis* | A | | |
| **Neoplecostominae** | *Pareiorhaphis* | *cameroni* | A | | |
| **Neoplecostominae** | *Pareiorhaphis* | *eurycephalus* | C | | |
| **Neoplecostominae** | *Pareiorhaphis* | *hystrix* | A/C | | |
| **Neoplecostominae** | *Pareiorhaphis* | *parmula* | C | | |
| **Neoplecostominae** | *Pareiorhaphis* | *steindachneri* | A | | |
| **Neoplecostominae** | *Pareiorhaphis* | *vestigipinnis* | C | | |
| **Neoplecostominae** | *Pareiorhina* | *carrancas* | B | | |
| **Neoplecostominae** | *Pareiorhina* | *carrancas* | B | | |
| **Neoplecostominae** | *Pareiorhina* | *hyptiorhachis* | A | | |
| **Neoplecostominae** | *Pareiorhina* | *rudolphi* | A | | |
| **Neoplecostominae** | *Pseudotocinclus* | *juquiae* | A | | |
| **Neoplecostominae** | *Pseudotocinclus* | *tietensis* | B | | |
| **Hypoptopomatinae** | *Acestridium* | *discus* | D | | |
| **Hypoptopomatinae** | *Acestridium* | sp. 1 | D | | |
| **Hypoptopomatinae** | *Hypoptopoma* | *gulare* | D | | |
| **Hypoptopomatinae** | *Hypoptopoma* | *inexspectatum* | C | | |
| **Hypoptopomatinae** | *Hypoptopoma* | *inexspectatum* | C | | |
| **Hypoptopomatinae** | *Hypoptopoma* | *inexspectatum* | C | | |
| **Hypoptopomatinae** | *Hypoptopoma* | sp. 1 | D | | |
| **Hypoptopomatinae** | *Lampiella* | *gibbosa* | A | | |
| **Hypoptopomatinae** | *Otocinclus* | *arnoldi* | C | | |
| **Hypoptopomatinae** | *Otocinclus* | *cocama* | D | | |
| **Hypoptopomatinae** | *Otocinclus* | *flexilis* | A | | |
| **Hypoptopomatinae** | *Otocinclus* | *hoppei* | D | | |
| **Hypoptopomatinae** | *Otocinclus* | *hoppei* | D | | |
| **Hypoptopomatinae** | *Otocinclus* | *mariae* | D | | |
| **Hypoptopomatinae** | *Otocinclus* | *mariae* | D | | |
| **Hypoptopomatinae** | *Otocinclus* | *vittatus* | C/D | | |
| **Hypoptopomatinae** | *Oxyropsis* | *acutirostra* | D | | |
| **Hypoptopomatinae** | *Oxyropsis* | sp. 1 | D | | |
|  |  |  |  | | |
| **Total valid names for Hypoptopomatinae, Neoplecostominae and Otothyrinae** | | | |  | **184** |
| **Total number of ingroup valid names** | | | |  | **87** |
| **Total number of possible new species in the present work** | | | |  | **17** |
| **Percent of valid species names included in the present work** | | | |  | **48 %** |
