# Supplementary material for: Molecular Phylogeny and Biogeographic History of the Armored Neotropical Catfish Subfamilies Hypoptopomatinae, Neoplecostominae and Otothyrinae (Siluriformes: Loricariidae)
Source: PLoS One. 2014 Aug 22;9(8):e105564. doi: 10.1371/journal.pone.0105564 (PMC4141799; doi:10.1371/journal.pone.0105564)
Supplement: Table S3 — Primers used in the present study to amplify partial sequences of F-reticulon 4, 16S rRNA, cytochrome oxidase subunit I (COI) and cytochrome B (CytB). (DOC) [file pone.0105564.s003.doc]

**Supplementary Table 3.** Primers used in the present study to amplify partial sequences of F-reticulon 4, 16S rRNA, cytochrome oxidase subunit I (COI) and cytochrome B (CytB).

| **Region and Fragment Length** | **Name** | **References** | **Primer Sequence** |
| --- | --- | --- | --- |
| **F-reticulon 4** | Freticul4-D | Chiachio et al. [36] | 5’-AGG CTA ACT CGC TYT SGG CTT TG-3’ |
|  | Freticul4-R |  | 5’-GGC AVA GRG CRA ART CCA TCT C-3’ |
|  | Freticul4 D2 |  | 5’-CTT TGG TTC GGA ATG GAA AC-3’ |
|  | Freticul4 R2 |  | 5’-AAR TCC ATC TCA CGC AGG A-3’ |
|  | Freticul4 iR |  | 5’-AGG CTC TGC AGT TTC TCT AG-3’ |
| **16S rRNA** | 16Sar | Kocher et al. [57] | 5’-ACG CCT GTT TAT CAA AAA CAT-3’ |
|  | 16Sbr |  | 5’-CCG GTC TGA ACT CAG ATC ACG T-3’ |
| **COI** | FishF1 | Ward et al. [59] | 5’-TCA ACC AAC CAC AAA GAC ATT GGC AC-3’ |
|  | FishR1 |  | 5’-TAG ACT TCT GGG TGG CCA AAG AAT CA-3’ |
| **CytB** | L14841 | Oliveira et al. [58] | 5`-CCA TCC AAC ATC TCA GCA TGA TGA AA 3` |
|  | H15915b |  | 5`-AAC CTC CGA TCT TCG GAT TAC AAG AC 3` |
