# Supplementary material for: Molecular Phylogeny and Biogeographic History of the Armored Neotropical Catfish Subfamilies Hypoptopomatinae, Neoplecostominae and Otothyrinae (Siluriformes: Loricariidae)
Source: PLoS One. 2014 Aug 22;9(8):e105564. doi: 10.1371/journal.pone.0105564 (PMC4141799; doi:10.1371/journal.pone.0105564)
Supplement: Table S4 — Nucleotide substitution models for each partition evaluated in the software PartitionFinder [68] and used in the phylogenetic analyses. *These partitions were analyzed in one partition. (DOC) [file pone.0105564.s004.doc]

**Supplementary Table 4.** Nucleotide substitution models for each partition evaluated in the software PartitionFinder [68] and used in the phylogenetic analyses. *These partitions were analyzed in one partition.

| **Gene** | **Maximum Likelihood**  **RAxML Analysis** | **Bayesian Inference with**  **Mrbayes v.3.0** | **Molecular Clock Analysis**  **with Beast v.1.6.2** | **Bases** |
| --- | --- | --- | --- | --- |
| **COI first base of codon** | GTR+I+G | GTR+I+G | GTR+I+G | 1 – 534 \3 |
| **COI second base of codon** | GTR+I+G | SYM+I+G | TrNef+I+G | 2 – 534 \3 |
| **COI third base of codon** | GTR+I | GTR+I | GTR+I | 3 – 534 \3 |
| **CytB first base of codon** | GTR+I+G | SYM+I+G | SYM+I+G | 535 – 1325 \3 |
| **CytB second base of codon** | GTR+I+G | GTR+I+G | GTR+I+G | 536 – 1325 \3 |
| **CytB third base of codon** | GTR+G | GTR+G | GTR+G | 537 – 1325 \3 |
| **16S** | GTR+I+G | GTR+I+G | GTR+I+G | 1326 – 1849 |
| **F-reticulon intron 1** | GTR+G | GTR+G | GTR+G | 1850 – 3274 |
| **F-retex2 first base of codon** | GTR+G | SYM+G | SYM+G | 3275 – 3536 \3 |
| ***F-retex2 second base of codon** | GTR+G | SYM+G | SYM+G | 3276 – 3536 \3 |
| ***F-retex2 third base of codon** | GTR+G | GTR+G | GTR+G | 3277 – 3536 \3 |
| **F-reticulon intron 2** | GTR+G | GTR+G | GTR+G | 3538 – 3881 |
| **F-retex3 first base of codon** | GTR+I+G | SYM+I+G | SYM+I+G | 3882 – 4500 \3 |
| **F-retex3 second base of codon** | GTR+I+G | GTR+I+G | GTR+I+G | 3883 – 4500 \3 |
| **F-retex3 third base of codon** | GTR+I+G | GTR+I+G | GTR+I+G | 3884 – 4500 \3 |
