# Supplementary material for: Molecular Phylogeny and Biogeographic History of the Armored Neotropical Catfish Subfamilies Hypoptopomatinae, Neoplecostominae and Otothyrinae (Siluriformes: Loricariidae)
Source: PLoS One. 2014 Aug 22;9(8):e105564. doi: 10.1371/journal.pone.0105564 (PMC4141799; doi:10.1371/journal.pone.0105564)
Supplement: Table S5 — DEC models tested to estimate distribution ranges inherited by the descending lineages at each node of the tree. The differences between the models are in the rate of dispersal among adjacent and no adjacent areas. * Represent the model used in the analysis. (DOC) [file pone.0105564.s005.doc]

**Supplementary Table 5.** DEC models tested to estimate distribution ranges inherited by the descending lineages at each node of the tree. The differences between the models are in the rate of dispersal among adjacent and no adjacent areas. * Represent the model used in the analysis.

|  | **Prediction** | **Dispersal rates between adjacent areas** | **Dispersal rates between no adjacent areas** | **Likelihood** |
| --- | --- | --- | --- | --- |
| M1 | Dispersal between no adjacent areas not permitted | 1.0 | − | lnL = - 255.9 |
| M2 | Dispersal between no adjacent areas permitted | 1.0 | 1.0 | lnL = - 254.8 |
| *M3 | Dispersal between no adjacent areas permitted | 0.5 | 0.0001 | lnL = - 252.5 |
| M4 | Dispersal between no adjacent areas permitted | 0.1 | 0.0001 | lnL = - 263.0 |
