# Supplementary material for: Molecular Phylogeny and Biogeographic History of the Armored Neotropical Catfish Subfamilies Hypoptopomatinae, Neoplecostominae and Otothyrinae (Siluriformes: Loricariidae)
Source: PLoS One. 2014 Aug 22;9(8):e105564. doi: 10.1371/journal.pone.0105564 (PMC4141799; doi:10.1371/journal.pone.0105564)
Supplement: Table S6 — Substitution Saturation estimated for each gene using the index of substitution saturation (Iss) [65], [66] and the rate of transitions/transversions evaluated in software DAMBE 5.2.31 [67]. (DOC) [file pone.0105564.s006.doc]

**Supplementary Table 6.** Substitution Saturation estimated for each gene using the index of substitution saturation (Iss) [65, 66] and the rate of transitions/transversions evaluated in software DAMBE 5.2.31 [67].

|  | **Model** | **Transition** | **Transversion** | **Index of Substitution Saturation (Iss)** |
| --- | --- | --- | --- | --- |
| **COI** | TN93 | 0.769 | 0.754 | Iss<Iss.c |
| **CytB** | GTR | 0.783 | 0.850 | Iss<Iss.c |
| **16S** | GTR | 0.914 | 0.844 | Iss<Iss.c |
| **Reticulon** | GTR | 0.937 | 0.950 | Iss<Iss.c |
