# Supplementary material for: Molecular Phylogeny and Biogeographic History of the Armored Neotropical Catfish Subfamilies Hypoptopomatinae, Neoplecostominae and Otothyrinae (Siluriformes: Loricariidae)
Source: PLoS One. 2014 Aug 22;9(8):e105564. doi: 10.1371/journal.pone.0105564 (PMC4141799; doi:10.1371/journal.pone.0105564)
Supplement: Table S7 — Likelihood-based tests for alternative topologies. SH and AU are probability values obtained for the Shimodaira-Hasegawa and the Approximately Unbiased tests [76]. Asterisks denote significant values (P<0.05 for SH and P<0.01 for AU and ELW), that imply the topology is rejected. (DOC) [file pone.0105564.s007.doc]

**Supplementary Table 7.** Likelihood-based tests for alternative topologies. SH and AU are probability values obtained for the Shimodaira-Hasegawa and the Approximately Unbiased tests [76]. Asterisks denote significant values (P<0.05 for SH and P<0.01 for AU and ELW), that imply the topology is rejected.

| **Test** | **Topology** | **- Ln *L*** | **∆ - Ln *L*** | **ELW** | **SH** | **AU** |
| --- | --- | --- | --- | --- | --- | --- |
|  | ML | 76971.76 |  |  |  |  |
| **1** | Monophyletic *Pareiorhaphis* | 76957.87 | 13.89 | 1 | 1 | 0.23 |
| **2** | Hypoptopomatinae sister group of Otothyrinae ͣ | 77001.46 | 29.70 | 0.0021* | 0.4540 | <0.001* |
| **3** | *Epactionotus* and *Eurycheilichthys* sister group of *Hisonotus* from South of Brazil ͣ | 77010.47 | 38.71 | 0.0089* | 0.3299 | 0.0136 |
| **4** | *Pareiorhina* genus monophyletic ͣ | 77102.71 | 130.95 | <0.001* | 0.0025* | <0.001* |
| **5** | *Neoplecostomus* genus monophyletic ͣ | 77120.75 | 148.99 | <0.001* | 0.0027* | <0.001* |
| **6** | Monophyletic *Pareiorhina* sister group to monophyletic *Neoplecostomus* | 77322.56 | 350.80 | <0.001* | <0.001* | <0.001* |
| **7** | Relationship of Otothyrinae genus consistent with Clade B of Schaefer (1991), Fig. 13 ͣ | 77523.21 | 551.45 | <0.001* | <0.001* | <0.001* |

ͣ The alternative topology was defined as the ML tree forcing the desired relationship.
